# Who Is Supplied With In‐Bed Sleepers (Pēpi‐Pod and Wahakura) for Reducing SUDI in New Zealand?

**DOI:** 10.1111/jpc.70136

**Published:** 2025-07-08

**Authors:** Edwin A. Mitchell, Stephanie Cowan, Jessica Wilson, John Thompson

**Affiliations:** ^1^ Department of Paediatrics: Child and Youth Health The University of Auckland Auckland New Zealand; ^2^ Change for Our Children Christchurch New Zealand

**Keywords:** cross‐sectional study, in‐bed sleeper, Pepi‐pod, sudden infant death, wahakura

## Abstract

**Aims:**

Smoking during pregnancy when combined with bedsharing is a major risk factor for Sudden Unexpected Death in Infancy (SUDI). In‐bed sleepers like Pēpi‐Pod and wahakura provide a safe space for infants within adult beds. But the distribution and reach of these devices to high‐risk infants remain unclear. Therefore, this study aimed to assess who receives in‐bed sleepers and determine if they are reaching infants at the highest risk of SUDI.

**Methods:**

We conducted a retrospective cross‐sectional study combining sales data analysis of Pēpi‐Pod in‐bed sleepers with data from the National Maternity Collection, Ministry of Health and Change for our Children from 2019 to 2021. We compared characteristics of infants receiving in‐bed sleepers with all New Zealand births.

**Results:**

An estimated 15.9% of all infants born in New Zealand during 2019–2021 received in‐bed sleepers, with significant regional variations. Only 37.5% of infants that were exposed to smoking during pregnancy received an in‐bed sleeper. Of in‐bed sleepers supplied, 72.9% were supplied to infants not exposed to smoking in pregnancy. In‐bed sleeper reporting was poor, with only 36.4% of those supplied being reported.

**Conclusion:**

The distribution of in‐bed sleepers is not optimally targeted, consistently failing to reach high‐risk infants, particularly those exposed to smoking in pregnancy. Therefore, improved strategies for distribution and reporting are necessary to enhance the effectiveness of this SUDI prevention measure.

**Summary**

- What is already known on this topic?
  ○ Exposure to maternal smoking in pregnancy in combination with bed sharing is a major risk factor for Sudden Unexpected Death in Infancy (SUDI).
  ○ In‐bed sleepers like Pēpi‐Pod and wahakura provide a safe space for an infant within an adult bed.
  ○ Supplying in‐bed sleepers to infants assessed as at higher SUDI risk was associated with a 29% decrease in post‐perinatal (7 days of life through to the first birthday) mortality from 2009 to 2015 (2.8 to 2.0/1000 live births).
- What this paper adds?
  ○ Sales data of Pēpi‐Pod in‐bed sleepers suggest 12.1% of infants born in the study period were supplied. In total, we estimated 15.9% of infants received in‐bed sleepers (either Pēpi‐Pod or wahakura), with considerable regional variations.
  ○ Only 37.5% of infants exposed to smoking during pregnancy received an in‐bed sleeper. Of in‐bed sleepers supplied, 72.9% were supplied to infants not exposed to smoking in pregnancy.
  ○ We conclude that the distribution of in‐bed sleepers is not optimally targeted, failing consistently to reach high‐risk infants, particularly those exposed to smoking in pregnancy. This inequitable distribution may be contributing to the stagnation of post‐perinatal mortality rates.

## 1 Introduction

Sudden unexpected death in infancy (SUDI) includes sudden infant death syndrome (SIDS), unknown cause, and accidental suffocation and strangulation in bed. SUDI causes 40–50 deaths per annum in New Zealand (0.7/1000 live births), the majority of which are potentially preventable [1]. More than 50% of SUDI cases occur while directly sharing a sleeping surface (bed sharing) with another person, usually the mother, and the infant is not in an infant bed on that surface [2]. This is also seen in other countries [3, 4, 5]. Epidemiological studies have identified factors that increase the risk of death while directly bed sharing. The main factor is smoking in pregnancy [2, 3, 5]. Smoke‐exposed infants are at considerably higher risk than smokefree infants when directly bed sharing. Infants who are less than 3 months of age are at higher risk than older infants [2, 3, 5], as are pre‐term compared to full‐term infants [5]. In addition, infants sleeping on sofas or directly bed sharing with parents who have drunk alcohol or taken recreational drugs are at higher risk [4]. Furthermore, social vulnerability is also associated with a higher prevalence of bed sharing [6].

In New Zealand, in‐bed sleepers such as Pēpi‐Pod and wahakura have been developed as a practical solution for parents who share their sleeping space with their infants while prioritising safety [7, 8]. Wahakura are hand‐woven from harakeke (flax) and Pēpi‐Pod are a polypropylene equivalent. By providing a designated infant bed within the adult bed, these products aim to combine the benefits of closeness with essential safety features to make bed sharing safer for babies. They offer a close and protected place to sleep when other infant bed types are either unsuitable or unavailable. From 2018, the expanded supply of in‐bed sleepers has been part of New Zealand's infant health strategy.

Placing infants to sleep in in‐bed sleepers breaks the link between ‘smoking in pregnancy’ and ‘direct’ bed sharing and de‐escalates the heightened risk of SUDI from exposure to the combination of both factors. Babies are close to their parents, enabling easy access for breastfeeding and settling [9].

Supplying in‐bed sleepers to infants assessed as at higher SUDI risk was associated with a 29% decrease in post‐perinatal (7 days of life through to the first birthday) mortality from 2009 to 2015 (2.8 to 2.0/1000 live births) [10]. The fall was greatest for Māori infants and in regions with the most intensive programmes. This evidence led the government to fund an expansion of the programme, which allowed 15% of infants born in New Zealand to be supplied with an in‐bed sleeper.

The distribution of in‐bed sleepers had three intervention phases based on the intensity of supply: low, medium and high. The first two phases were community developments, the third a government expansion of these. The low intensity phase (< 250 in‐bed sleepers/year or < 4/1000 live births) was between 2006 and 2010 when weaving wahakura sleepers was developing in some Māori communities. The medium intensity phase (< 3000 in‐bed sleepers/year or < 50/1000 live births) was between (2011 and 2017) when Pēpi‐Pod sleepers, a polypropylene version, were introduced, enabling increased supply. The high intensity phase (> 8000 in‐bed sleepers/year or > 130/1000 live births) since 2018 started when the earlier developments were expanded by the Ministry of Health and embedded into infant health policy and practice.

The initial fall in post‐perinatal mortality associated with the introduction of in‐bed sleepers through community developments did not continue for the expanded programme.

**FIGURE 1:**
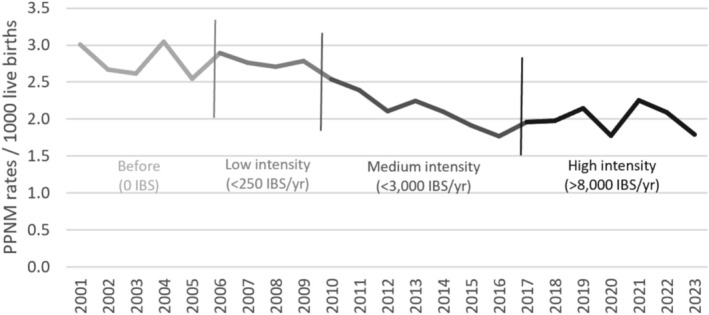
Annual post‐perinatal (1–52 weeks) mortality rates and in‐bed sleeper (IBS) intervention phases.

Figure 1 tracks these intervention phases against post‐perinatal mortality rates (Data source: Statistics NZ).

While the reasons for the failure to reduce mortality further are unknown, one possibility is poor targeting of resources to need. The aim of this study was to describe who is currently supplied with in‐bed sleepers. Our central hypothesis was that not enough high‐risk infants were receiving in‐bed sleepers for there to be a measurable impact on mortality.

## 2 Methods

### 2.1 Study Design

There are two components of this study. Firstly, we assessed the number of Pēpi‐Pod in‐bed sleepers purchased by each District Health Board (DHB) from sales data. There are no data for wahakura sales. Secondly, we undertook a retrospective cross‐sectional study. The characteristics of those families supplied with in‐bed sleepers over a three‐year period (2019–2021) were compared with the characteristics of all births in New Zealand over the same 3 years. We followed the Strengthening the Reporting of Observational Studies in Epidemiology (STROBE) reporting guideline for cross‐sectional studies.

### 2.2 Data Collection

#### 2.2.1 Study 1

Sales data were supplied by Change for our Children (CC) for 2019–2021. The number of births in each DHB was obtained from Statistics NZ. The percentage of infants supplied with Pēpi‐Pod in‐bed sleepers in each DHB was calculated.

#### 2.2.2 Study 2

Datasets were provided from three sources: the National Maternity Collection (MAT) dataset, the Ministry of Health (MoH) and CC. The MAT dataset provides statistical, demographic, and clinical information about selected publicly funded maternity services up to 9 months before and 3 months after a birth. MAT contains data on primary maternity services provided under Section 88 of the New Zealand Public Health and Disability Act 2000. MAT also contains inpatient and day‐patient health event data during pregnancy, birth and the postnatal period for mother and baby, sourced from the National Minimum Dataset.

The MoH requested DHBs to report on who were supplied with a range of infant bed types from January 2019 to December 2021. CC, who supplies the Pēpi‐Pod Programme, also requested reporting on who had been supplied with an in‐bed sleeper as part of programme monitoring.

These three datasets were merged using encrypted National Health Index number (NHI number) by the MoH. The NHI number is a unique identifier that is assigned to every person who uses health and disability support services in New Zealand. Duplicates were excluded. In addition, records that stated the infant bed supplied was a box (*n* = 2) and those where the in‐bed sleeper was offered but not accepted (*n* = 3) were also excluded.

### 2.3 Variable Definitions

Maternal variables: mother's age categorised as < 20, 20–24, 25–29, 30–34, 35+, unknown/missing years; mother's ethnicity: Māori, Pacific, Asian, NZ European and Other, unknown/missing; parity: 0, 1, 2, 3, 4, 5+, unknown/missing; maternal smoking at registration with the mother's lead maternity carer (LMC): yes, no, unknown/missing; socioeconomic status was assessed using New Zealand Index of Deprivation (NZDep). The NZDep is an area‐based measure of socioeconomic deprivation in New Zealand [11]. It measures the level of deprivation for people in each small area. It is based on nine Census variables. NZDep groups the deprivation scores into quintiles, where one represents the areas with the least deprived scores and five the areas with the most deprived scores.

Infant variables: sex: male, female, unknown/missing; plurality: singleton, multiple/twin, unknown/missing; birthweight (g): < 2500, 2500–2999, 3000–3499, 3500–3999, 4000+, unknown/missing; gestational age (completed weeks): < 37, 37+, unknown/missing; breastfeeding at discharge from obstetric hospital: artificial, partial, exclusive/fully, unknown/missing.

### 2.4 Statistical Analysis

Odds ratios (OR) and their 95% confidence intervals (CI) were used to compare the factors that determine the distribution of in‐bed sleepers. All analyses used SAS software Package v9.4 (SAS Institute).

## 3 Results

### 3.1 Study 1

There were 175 863 babies born over the 3‐year study period which occurred in the ‘high intensity’ intervention phase. Table 1 shows the number of Pēpi‐Pod beds bought by each DHB, the number of births in each DHB and the percentage of these births that were supplied with Pēpi‐Pod beds. Overall, 21 288 Pēpi‐Pod in‐bed sleepers were purchased during the 3‐year study period, enabling 12.1% of infants born to have received one (Table 1). Coverage varied considerably with four regions supplying Pēpi‐Pod beds for more than 20% of infants born in their DHBs, whereas at the lower end of supply, four DHBs covered less than 6% of their infants.

**TABLE 1:** The number of Pēpi‐Pod in‐bed sleepers purchased, the reported number of Pēpipod and wahakura supplied and the estimated total number of in‐bed sleepers supplied for each District Health Board.

	Total births	Pēpi‐Pod beds purchased by DHB		Pepi‐pod		Wahakura			Estimated total of IBS	
		Number	% of births	# reported	% of purchase	# reported	Estimated #	% of births	Estimated #	% of births
Auckland	15 498	1235	8.0	231	18.7	25	134	0.9	1369	8.8
Bay of Plenty	9393	1266	13.5	636	50.2	64	127	1.4	1393	14.8
Canterbury	19 287	3774	19.6	1465	38.8	457	1177	6.1	4951	25.7
Capital & Coast	9486	364	3.8	129	35.4	195	550	5.8	914	9.6
Counties Manukau	24 879	3934	15.8	1479	37.6	140	372	1.5	4306	17.3
Hawke's Bay	6240	1029	16.5	149	14.5	98	677	10.8	1706	27.3
Hutt Valley	5979	153	2.6	47	30.7	198	645	10.8	798	13.3
Lakes	4380	1027	23.4	122	11.9	23	194	4.4	1221	27.9
MidCentral	6552	604	9.2	87	14.4	79	548	8.4	1152	17.6
Nelson‐Marlborough	4389	434	9.9	215	49.5	99	200	4.6	634	14.4
Northland	6759	654	9.7	260	39.8	242	609	9.0	1263	18.7
South Canterbury	1803	216	12.0	109	50.5	3	6	0.3	222	12.3
Southern	10 206	1134	11.1	684	60.3	94	156	1.5	1290	12.6
Tairawhiti	2016	422	20.9	123	29.1	21	72	3.6	494	24.5
Taranaki	4506	86	1.9	89	103.5	313	302	6.7	388	8.6
Waikato	16 596	2561	15.4	893	34.9	87	250	1.5	2811	16.9
Wairarapa	1590	330	20.8	207	62.7	12	19	1.2	349	22.0
Waitemata	22 878	1468	6.4	406	27.7	24	87	0.4	1555	6.8
West Coast	1002	56	5.6	51	91.1	5	5	0.5	61	6.1
Whanganui	2424	541	22.3	322	59.5	266	447	18.4	988	40.8
Overseas				2		1			0	
Missing				40		11			0	
TOTAL	175 863	21 288	12.1	7746	36.4	2457	6752	3.8	28 040	15.9

### 3.2 Study 2

There were 10 229 individual reports to MoH or CC. Of these, 7746 (75.7%) Pēpi‐Pod beds were supplied, 2457 (24.0%) wahakura, and 26 (0.3%) were not specified. Overall, the reporting mechanism only detected 36.4% (7746/21 288) of Pēpi‐Pod beds purchased by the DHBs. The level of reporting varied considerably by DHB, ranging from 11.9% to 103.5%. There was a significant correlation between reported distribution of Pēpi‐Pod beds and their purchases by DHBs (*r* = 0.95, *p* < 0.0001).

The total number of wahakura supplied was estimated from the number of wahakura reportedly distributed by each DHB and considering the level of under‐reporting by each DHB. Overall, the estimated supply of wahakura was 3.8% of all births and varied by DHB from less than 1% of births to over 10% of births.

Pēpi‐Pod sales and estimated number of wahakura were added to estimate the total in‐bed sleeper supply. This was 15.9% of all births. Again, this varied considerably by DHB from 6.1% to 40.8%.

Table 2 shows the characteristics of the total population (from MAT dataset), those who were reported to have been supplied with an in‐bed sleeper (MoH and Change for our Children data) and those who were estimated to have been supplied with an in‐bed sleeper.

**TABLE 2:** Characteristics of infants and their families for those supplied in‐bed sleepers and the odds (95% confidence intervals) of being supplied with an in‐bed sleeper.

			In‐bed sleeper provided				Estimated in‐bed sleeper provided					
	Total population		No		Yes		No		Yes			
	*N* = 179 007		*N* = 168 778		*N* = 10 229		*N* = 151 158		*N* = 27 849		Univariable	
	*N*	%	*N*	%	*N*	%	*N*	%	*N*	%	OR	(95% CI)
MOTHER AGE												
< 20	5750	3.2	4858	2.9	892	8.7	3321	2.2	2429	8.7	3.90	(3.68–4.13)
20–24	24 756	13.8	22 372	13.3	2384	23.3	18 265	12.1	6491	23.3	1.90	(1.83–1.97)
25–29	48 520	27.1	45 706	27.1	2814	27.5	40 859	27.0	7661	27.5	Ref	
30–34	60 913	34.0	58 318	34.6	2595	25.4	53 848	35.6	7065	25.4	0.70	(0.68–0.72)
35+	39 059	21.8	37 516	22.2	1543	15.1	34 858	23.1	4201	15.1	0.64	(0.062–0.67)
Unknown/missing	9		8		1		6		3			
MOTHER_ETHNIC_GROUP									27 849			
Maori	45 473	25.4	40 495	24.0	4978	48.7	31 920	21.1	13 553	48.7	4.94	(4.79–5.11)
Pacific	18 115	10.1	16 555	9.8	1560	15.3	13 868	9.2	4247	15.3	3.57	(3.42–3.72)
Asian	34 507	19.3	33 167	19.7	1340	13.1	30 859	20.4	3648	13.1	1.38	(1.32–1.44)
NZ European/Other	80 885	45.2	78 535	46.5	2350	23.0	74 487	49.3	6398	23.0	Ref	
Unknown/missing	27		26		1		24		3			
INFANT SEX									27 849			
M	91 656	51.2	86 321	51.2	5335	52.2	77 131	51.0	14 525	52.2	1.05	(1.02–1.07)
F	87 301	48.8	82 415	48.8	4886	47.8	73 998	49.0	13 303	47.8	Ref	
Unknown/missing	50		42		8		28		22			
PARITY						0.0						
0	72 054	41.7	67 836	41.6	4218	43.8	60 570	41.3	11 484	43.8	Ref	
1	57 493	33.3	55 101	33.8	2392	24.9	50 981	34.8	6512	24.9	0.67	(0.65–0.70)
2	24 822	14.4	23 476	14.4	1346	14.0	21 157	14.4	3665	14.0	0.91	(0.88–0.95)
3	10 075	5.8	9283	5.7	792	8.2	7919	5.4	2156	8.2	1.43	(1.36–1.51)
4	4359	2.5	3911	2.4	448	4.7	3139	2.1	1220	4.7	2.05	(1.91–2.20)
5+	3914	2.3	3490	2.1	424	4.4	2760	1.9	1154	4.4	2.21	(2.05–2.37)
Unknown/missing	6290		5681		609		4632		1658			
BIRTHWEIGHT												
< 2500	10 864	6.4	9636	6.0	1228	12.5	7521	5.2	3343	12.5	2.56	(2.45–2.69)
2500–2999	25 127	14.7	23 362	14.5	1765	17.9	20 322	14.1	4805	17.9	1.36	(1.31–1.41)
3000–3499	57 074	33.4	53 977	33.5	3097	31.4	48 642	33.8	8432	31.4	Ref	
3500–3999	53 362	31.2	50 781	31.5	2581	26.2	46 335	32.2	7027	26.2	0.87	(0.85–0.91)
4000+	24 431	14.3	23 252	14.4	1179	12.0	21 221	14.7	3210	12.0	0.87	(0.84–0.91)
Unknown/missing	8149		7770		379		7117		1032			
PLURALITY												
Singleton	173 921	97.4	164 236	97.6	9685	95.1	147 553	97.8	26 368	95.1	Ref	
Multiple/twin	4603	2.6	4104	2.4	499	4.9	3244	2.2	1359	4.9	2.34	(2.20–2.50)
Unknown/missing	483		438		45		360		123			
BREASTFEEDING AT DISCHARGE												
Artificial	17 668	11.2	16 322	10.9	1346	15.9	14 003	10.4	3665	14.3	1.49	(1.43–1.55)
Partial	30 013	19.0	28 117	18.8	1896	22.3	24 851	18.4	5162	20.1	1.18	(1.14–1.22)
Exclusive/fully	110 219	69.8	104 977	70.3	5242	61.8	95 948	71.2	16 885	65.7	Ref	
Unknown/missing	21 107		19 362		1745		16 356		4751			
SMOKING STATUS AT FIRST LMC REGISTRATION												
N	153 769	89.1	146 760	90.0	7009	72.9	134 686	91.9	19 083	72.9	Ref	
Y	18 888	10.9	16 288	10.0	2600	27.1	11 809	8.1	7079	27.1	4.23	(4.09–4.37)
Unknown/missing	6350		5730		620		4662		1688			
GESTATIONAL AGE												
< 37	13 861	7.8	12 388	7.3	1473	14.4	9851	6.5	4010	14.4	2.42	(2.32–2.51)
37+	164 980	92.2	156 241	92.7	8739	85.6	141 187	93.5	23 793	85.6	Ref	
Unknown/missing	166		149		17		120		46			
NZ DEP INDEX QUINTILE												
1 Least deprived	27 465	15.4	26 534	15.8	931	9.2	24 930	16.6	2535	9.2	Ref	
2	29 465	16.5	28 403	16.9	1062	10.4	26 574	17.7	2891	10.4	1.07	(1.01–1.13)
3	32 477	18.2	31 044	18.5	1433	14.1	28 576	19.0	3901	14.1	1.34	(1.27–1.42)
4	38 609	21.7	36 159	21.5	2450	24.1	31 939	21.2	6670	24.1	2.05	(1.96–2.16)
5 Most deprived	50 129	28.1	45 832	27.3	4297	42.2	38 430	25.5	11 699	42.2	2.99	(2.86–3.13)
Missing	862		806		56		710		152			
RISK LEVEL CATERGORIES: HIGH VS LOW RISK												
High	26 846	15.0	23 285	13.8	3561	34.8	17 151	11.3	9695	34.8	4.17	(4.05–4.30)
Low	152 161	85.0	145 493	86.2	6668	65.2	134 007	88.7	18 154	65.2	Ref	

The following factors were associated with being supplied with an in‐bed sleeper:

- Maternal factors: young, Māori, Pacific and Asian ethnicities, smoking, living in areas of socioeconomic deprivation
- Obstetric factors: parity 3+, and multiple pregnancy, and
- Infant factors: male, birthweight < 3000 g, preterm birth and bottle fed.

The key variable is exposure to smoking in pregnancy. The MAT dataset indicates 18 888 (10.9%) of infants born in the 3‐year study period were exposed to smoking during pregnancy. Of in‐bed sleepers supplied, 72.9% were supplied to non‐exposed infants; thus, there was more than sufficient supply to all smoke‐exposed infants.

## 4 Discussion

Sales data suggests 12.1% of infants get Pēpi‐Pod in‐bed sleepers and we have estimated 3.8% of infants get wahakura. Thus, 15.9% of infants are supplied with in‐bed sleepers. At first glance, in‐bed sleepers are going to the right families. Infants exposed to smoking in pregnancy are more likely to be supplied with in‐bed sleepers than those who are not exposed. However, in terms of numbers, most (62.5%) infants who were smoke‐exposed in pregnancy were not supplied with in‐bed sleepers. In fact, 72.9% of in‐bed sleepers went to smokefree infants. While there may be other reasons for supplying an in‐bed sleeper to an infant, such as prematurity or low birthweight, it is infants exposed to smoking in pregnancy who should be prioritised, as this is the major factor that escalates the risk of death in combination with direct bed sharing [2].

Limitations of this study need to be considered. The first point is that monitoring of in‐bed sleeper distribution is poor. Only about a third of Pēpi‐Pod in‐bed sleepers purchased were reported to MoH or CC as being distributed to specific families. A consequence is that we are estimating relative risks based on only a proportion (36.4%) of those supplied with in‐bed sleepers. This lack of comprehensive reporting creates uncertainty in the data and limits the accuracy of the conclusions. However, it seems likely that the failure to report was random, as there was a significant positive correlation between purchase of Pēpi‐Pods beds and reports of distribution to MoH and CC. Secondly, there are no sales data for wahakura. Wahakura may be woven by pregnant women or their whānau (families) in the community, and these would not be captured by the reporting mechanism. Furthermore, Pēpi‐Pod beds are designed for reuse within communities, and families are encouraged to share their in‐bed sleepers with other families when no longer required, and this on‐use is not captured.

In the two community‐based phases of the intervention, the impact on post‐perinatal mortality was as expected based on the strength of evidence of an interaction between smoking in pregnancy and bedsharing risks, and an increasing supply of in‐bed sleepers. A failure to continue impact into the high intensity phase could also be due to changes made in adapting the intervention and its core elements for expansion [12]. This has not been studied or reported for this program.

Clarity is needed about what an in‐bed sleeper intervention is. It is specific to breaking the link between smoking in pregnancy and bedsharing and carries the best single chance for protecting infants from SUDI. Definition of a previously used term ‘safe sleep device’ (SSD) has been expanded by others to include many infant bed types such as cots, portacots, bassinets, Moses baskets etcetera [13]. However, these products will not enable indirect bed sharing. An in‐bed sleeper intervention will.

The findings of this study lead to the following recommendations. Firstly, monitoring of the distribution of in‐bed sleepers needs to improve, occur at central level, and be reported on in a timely fashion. These data were from 2019 to 2021, then collection was stopped by MoH. The key objective of monitoring and surveillance in public health is to provide information to guide interventions and improve accuracy of implementation; in this study, accuracy means distribution to higher numbers of high‐risk infants, especially infants exposed to smoking in pregnancy.

Secondly, the distribution of in‐bed sleepers varies significantly across regions. This is an example of postcode lottery which refers to variations in health care between different geographical areas that appears arbitrary and unlinked to health need [14]. To address this inequity, we recommend tighter criteria for in‐bed sleeper distribution where access is based on the risk‐exposure of infants, is independent of region, and nationally coordinated. For example, ‘intention to bed share’ is not a criterion. ‘Smoke‐exposed in pregnancy’ is. Ideally, where a targeted rather than universal approach is taken, in‐bed sleepers would be distributed to those at highest risk, such as to infants who were smoke‐exposed in pregnancy, as this information is collected routinely and is the factor that escalates SUDI risk from small to substantial when a baby is directly bedsharing. Families with infants at lower risk of SUDI may choose to arrange their own in‐bed sleeper.

We propose use of the term ‘in‐bed sleeper’ to describe collectively infant beds such as Pēpi‐Pod and wahakura and recommend that bed sharing be defined as direct (infant not in an in‐bed sleeper) or indirect (infant in an in‐bed sleeper) in future studies.

In conclusion, there is considerable variation in in‐bed sleeper distribution rates by DHB. Furthermore, approximately three quarters of in‐bed sleepers appear to be distributed to infants that are at low risk of SUDI, and over 60% of infants exposed to smoking in pregnancy miss out. This is likely a factor in why post‐perinatal mortality rates have not reduced further during the high intensity phase and may even be increasing. In‐bed sleepers were designed specifically to break the link between smoking in pregnancy and bed sharing—a combination that otherwise results in escalating SUDI risk. In‐bed sleepers need to be used by high‐risk infants when bed sharing if SUDI mortality rates are to be lowered.

## Ethics Statement

This study was approved by the Auckland Health Research Ethics Committee (AHREC) Reference number 24728 entitled ‘Who is supplied with Pepi‐pods and wahakura’.

## Conflicts of Interest

Stephanie Cowan developed the Pēpi‐Pod Program (PPP) is Director of Change for our Children and as such has a professional interest in the reputation of the programme. Change for our Children supplies the Pēpi‐Pod Program on a self‐funded, cost recovery basis (i.e., as a social business), owns the intellectual property and service mark of the PPP, and pays a salary to Stephanie Cowan. These things constitute a commercial interest. The sole source of funds for providing the PPP comes from the sale of goods and services associated with it. There is no funding from any other source. The other authors declare no conflicts of interest.
